# The urethral position may shift due to urethral catheter placement in the treatment planning for prostate radiation therapy

**DOI:** 10.1186/s13014-019-1424-8

**Published:** 2019-12-12

**Authors:** Yasuhiro Dekura, Kentaro Nishioka, Takayuki Hashimoto, Naoki Miyamoto, Ryusuke Suzuki, Takaaki Yoshimura, Ryuji Matsumoto, Takahiro Osawa, Takashige Abe, Yoichi M. Ito, Nobuo Shinohara, Hiroki Shirato, Shinichi Shimizu

**Affiliations:** 1 Department of Radiation Oncology, Graduate School of Medicine, Hokkaido University, North-15, West-7, Kita-Ku, Sapporo, Hokkaido 060-8638 Japan; 2 Department of Radiation Medical Science and Engineering, Faculty of Medicine, Hokkaido University, North-15, West-7, Kita-Ku, Sapporo, Hokkaido 060-8638 Japan; 3 Department of Radiation Medicine, Faculty of Medicine, Hokkaido University, North-15, West-7, Kita-Ku, Sapporo, Hokkaido 060-8638 Japan; 4 Global Station for Biomedical Science and Engineering, Global Institute for Cooperative Research and Education, Hokkaido University, North-15, West-7, Kita-Ku, Sapporo, Hokkaido 060-8638 Japan; 5 Department of Medical Physics, Hokkaido University Hospital, North-14, West-5, Kita-Ku, Sapporo, Hokkaido 060-8638 Japan; 6 Department of Health Sciences and Technology, Faculty of Health Sciences, Hokkaido University, North-12, West-5, Kita-Ku, Sapporo, Hokkaido 060-0812 Japan; 7 Department of Renal and Genitourinary Surgery, Faculty of Medicine, Hokkaido University, North-15, West-7, Kita-Ku, Sapporo, Hokkaido 060-8638 Japan; 8 Department of Statistical Data Science, The Institute of Statistical Mathematics, 10-3, Midori-cho, Tachikawa, Tokyo 190-0014 Japan

**Keywords:** Prostate cancer, Radiotherapy, Urethra

## Abstract

**Purpose:**

To determine the best method to contour the planning organ at risk volume (PRV) for the urethra, this study aimed to investigate the displacement of a Foley catheter in the urethra with a soft and thin guide-wire.

**Methods:**

For each patient, the study used two sets of computed tomography (CT) images for radiation treatment planning (RT-CT): (1) set with a Foley urethral catheter (4.0 mm diameter) plus a guide-wire (0.46 mm diameter) in the first RT-CT and (2) set with a guide-wire alone in the second CT recorded 2 min after the first RT-CT. Using three fiducial markers in the prostate for image fusion, the displacement between the catheter and the guide-wire in the prostatic urethra was calculated. In 155 consecutive patients treated between 2011 and 2017, 5531 slices of RT-CT were evaluated.

**Results:**

Assuming that ≥3.0 mm of difference between the catheter and the guide-wire position was a significant displacement, the urethra with the catheter was displaced significantly from the urethra with the guide-wire alone in > 20% of the RT-CT slices in 23.2% (36/155) of the patients. The number of patients who showed ≥3.0 mm anterior displacement with the catheter in ≥20% RT-CT slices was significantly larger at the superior segment (38/155) than at the middle (14/155) and inferior segments (18/155) of the prostatic urethra (*p* < 0.0167).

**Conclusions:**

The urethral position with a Foley catheter is different from the urethral position with a thin and soft guide-wire in a significant proportion of the patients. This should be taken into account for the PRV of the urethra to ensure precise radiotherapy such as in urethra-sparing radiotherapy.

## Background

**Fig. 1:**
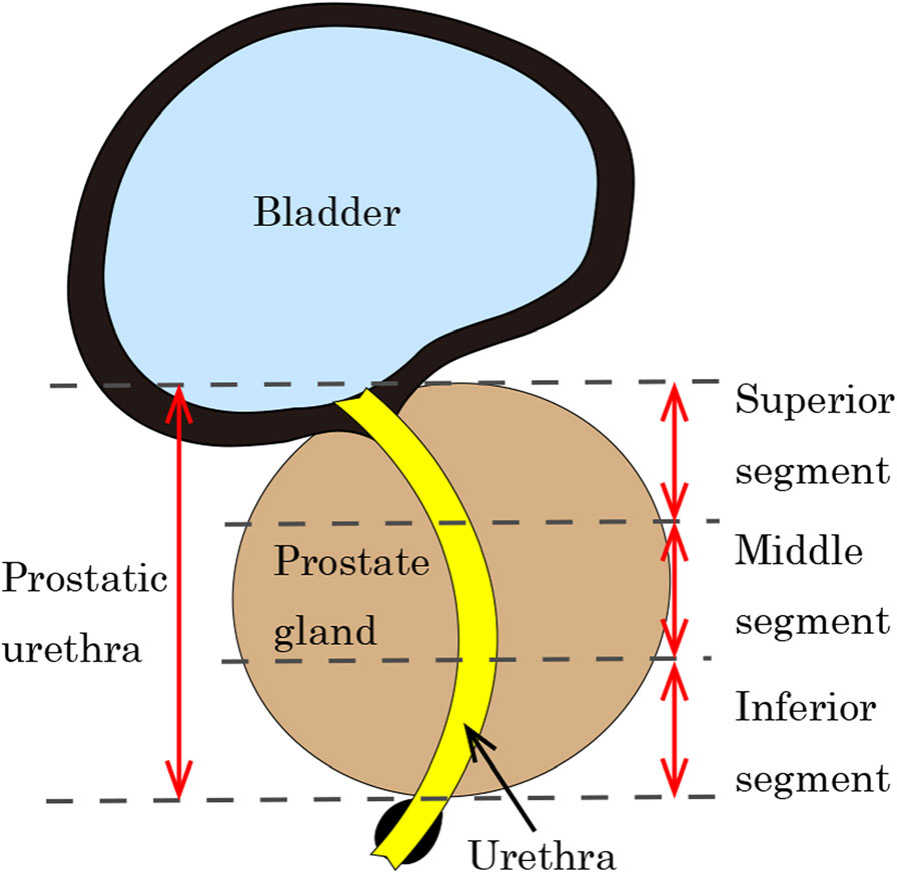
Illustration of the normal anatomy and three segments of prostatic urethra as defined and used in this study. The prostatic urethra is defined as the urethra in the prostate, i.e., from the bladder to the prostate/pelvic floor border, as shown on computed tomography. The superior segment is defined one-third of all prostatic urethra to the bladder side, and the inferior segment is the portion to the pelvic floor side. Each segment is divided evenly

Radiation therapy is a common treatment modality for localized prostate cancer [1, 2]. The image-guided intensity-modulated radiotherapy (IG-IMRT) with either conventional or hypofractionated schedules is now the standard technology, and the incidences of acute and late gastro-intestinal (GI) and genito-urinary (GU) toxicities are considerably lower than before [1–4]. Nowadays, the risk of GI toxicities in IG-IMRT is further reduced by advanced technology such as rectal spacer [5]. Although the incidence of GU toxicities is sufficiently low in recent studies [4, 6], a small proportion of patients still may experience GU toxicities [7, 8]. Recently, urethra-sparing radiotherapy (USRT) using IG-IMRT, in which the dose to the prostatic urethra (Fig. 1) is intentionally reduced, is attracting attention and reported as an attempt to minimize GU toxicities furthermore [9–12]. For example, Shimizu et al. have proposed USRT for prostate cancer [10] using fluoroscopic real-time tracking technology, called real-time tumor-tracking radiotherapy, and three fiducial gold markers with the accuracy of ±1–2 mm [13–18]. However, it is still not clear whether significant urethral sparing is feasible without deterioration in tumor control by IG-IMRT even if it would be desirable.

The essential technical problem to be solved in USRT is the identification of the urethra in the prostate gland when planning for radiation treatment [19]. Since the density of the urethral wall on computed tomography (CT) images is the same as the prostate gland, the geometric center of the prostate has been used as a “surrogate urethra” [20]. The position of the Foley catheter in the prostate gland has also been used as the basis of the urethral position in CT planning [21, 22]. However, there is also a concern that the Foley catheter may rotate or shift the physiological position of the urethra from the actual position of the “urethra without the catheter” [12, 21]. As a result, if a Foley catheter is used in CT for treatment planning but not in the actual delivery of USRT, there is a risk of introducing discrepancies in the urethral position and dose distribution between the planned treatment and the actual treatment. Magnetic resonance imaging (MRI) is expected to be useful for this purpose [23, 24], but the reported inter-observer variation in the male pelvic anatomy on MRI is not negligible [24].

Shimizu et al. have developed a unique CT planning method in this regard by contouring the urethra with a soft, thin guide-wire in addition to the Foley catheter in the treatment planning for USRT [10]. However, they did not provide further details of the method. Thus, the present study aimed to investigate the magnitude of the positional discrepancy between the urethra with the Foley catheter plus guide-wire and the urethra with only the guide-wire in the CT for treatment planning.

## Methods

### Image acquisition (Fig. 2)

1. Three gold fiducial markers (2.0 mm diameter) were inserted around the prostate gland, i.e., one at the apex of the prostate and two at the left and right of the base of the gland.

**Fig. 2:**
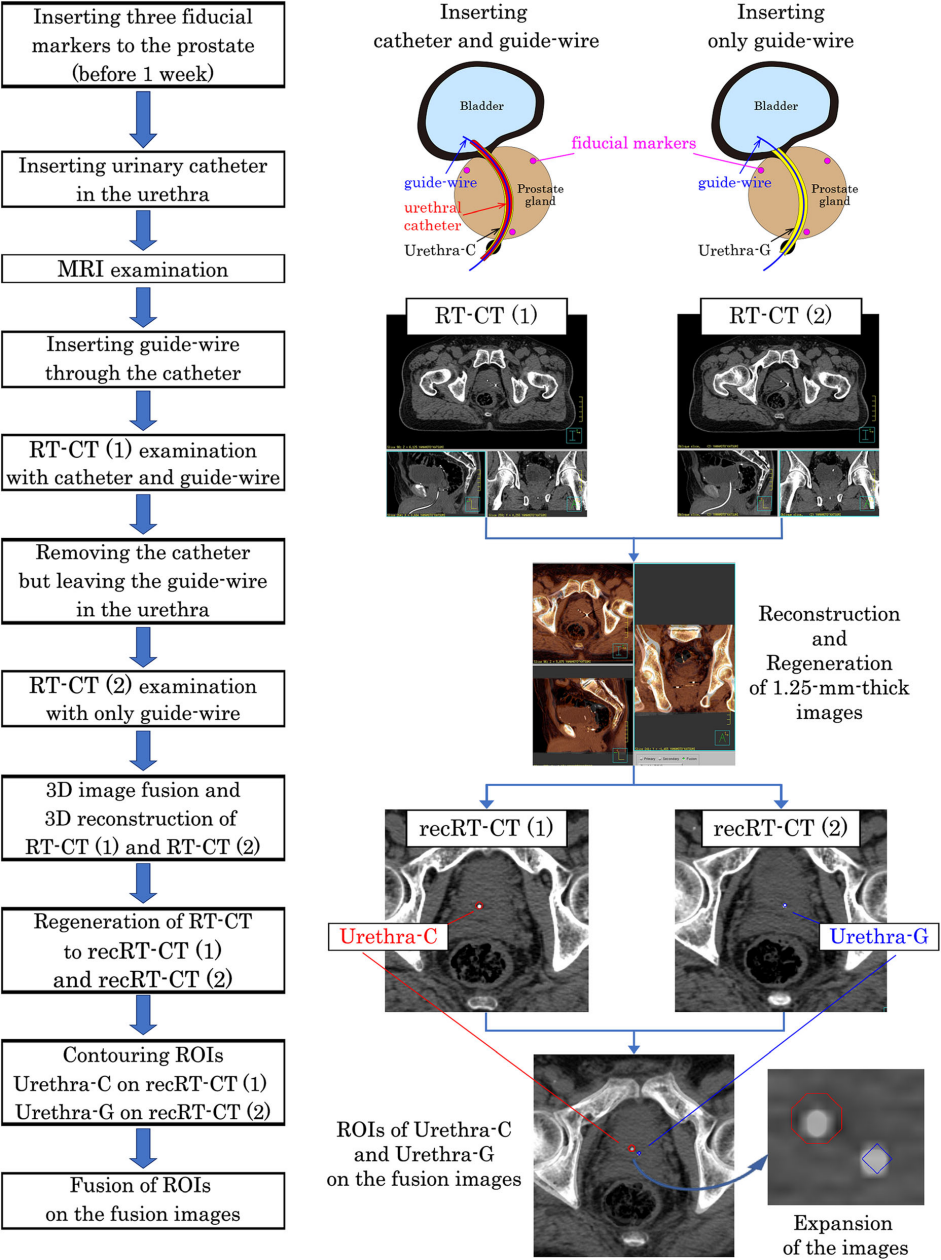
Flowchart of the image acquisition, contouring, and calculation. Flow chart of the procedures is shown in the left side. Representative images are shown in the right side for each procedure. Please see sections *Image acquisition* and *Contouring and Calculation* for details

One week later, MR and CT images were acquired for treatment planning with the following procedures.

2. A 12-French Foley catheter (4.0 mm outer diameter, Bardia® Biocath® Foley Catheter, Medicon Co., Tokyo) was inserted into the urethra after urination and defecation. After complete urination, 50 mL of sterile normal saline was introduced into the bladder.
3. MRI of the pelvis was performed to contour the prostate gland and other structures as the clinical target volume [25] with the urethral Foley catheter in place.
4. Following the MRI, a soft and thin guide-wire (0.46 mm diameter, Radifocus® Guide-wire M, TERUMO Co., Tokyo) was inserted into the urethral Foley catheter.
5. CT of the pelvis was performed with the guide-wire inside the urethral Foley catheter, and we called the CT image sets as RT-CT (1).
6. As the guide-wire was retained in the urethra, the outer sheath, i.e., urethral Foley catheter, was removed from the patient.
7. Another CT of the pelvis was performed only with the guide-wire remaining in the urethra, as the urethral Foley catheter was already removed. We called the CT image sets as RT-CT (2).

Each of the RT-CT images was obtained in 1.25-mm-thick slices of the whole prostate gland and seminal vesicles. The time interval between the start of RT-CT (1) and RT-CT (2) was set to 2 min for all patients.

### Contouring and calculation (Figs. 2 and 3)

The contouring was undertaken by the same radiation oncologist (YD) and checked by a different more senior radiation oncologist (KN) to maintain consistency.

**Fig. 3:**
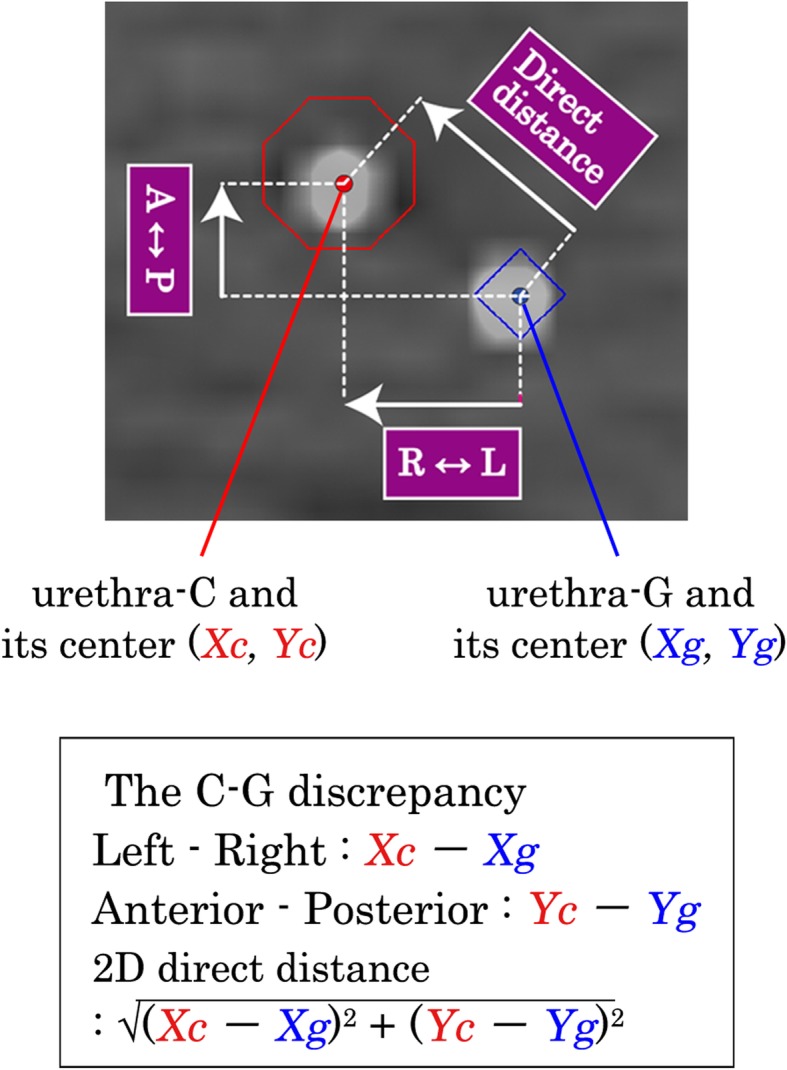
Illustration of the definition of “C-G discrepancy”. On the top image, the red point shows the center of urethra-C defined as coordinates *Xc* and *Yc,* and the blue point shows the center of urethra-G defined as coordinates *Xg* and *Yg*. The “C-G discrepancy” is calculated from the coordinates of the center of the ROI for urethra-C and urethra-G along the antero-posterior direction and the left-right direction. Two-dimensional (2D) direct distance between urethra-C and urethra-G are calculated using these distances. On the lower box, the definition of the “C-G discrepancy” is shown. R↔L, left-right direction; A↔P, anteroposterior direction; direct distance, 2D direct distance

1. Two sets of RT-CT images were then transferred to a treatment planning system, Pinnacle^3^ (Phillips Medical Systems, WI, USA).
2. Three-dimensional (3D) rigid image fusion and 3D reconstruction of RT-CT (1) and RT-CT (2) was performed using three fiducial markers to fit the coordinates of the two image sets.
3. Then, 1.25-mm-thick images from the reconstructed RT-CT (1) were regenerated, and we named the reconstructed RT-CT (1) as recRT-CT (1).
4. The catheter in the slices of recRT-CT (1) was contoured as a round region of interest (ROI) with 4 mm diameter assuming the guide-wire in the urethral catheter on the recRT-CT (1) as the center of the catheter, and we called this ROI as urethra-C.
5. Then, 1.25-mm-thick images from the reconstructed RT-CT (2) were regenerated, and we named the reconstructed RT-CT (2) as recRT-CT (2).
6. The guide-wire in the recRT-CT (2) was contoured as a round ROI with 2.0 mm diameter assuming the guide-wire on the recRT-CT (2) as the center of the guide-wire, and we called the ROI as urethra-G.
7. Two high-density spots of a guide-wire in the catheter in the recRT-CT (1), i.e., urethra-C, and a guide-wire in the recRT-CT (2), i.e., urethra-G, are visualized clearly in the fusion images.
8. The two-dimensional (2D) displacement to the center of the urethra-C from that of urethra-G was calculated on each slice of fusion images (Fig. 3). In this study, the difference in millimeters between urethra-C and urethra-G was called “C-G discrepancy”.

The C-G discrepancies in the coordinates along the anteroposterior (AP, ventral-dorsal) direction, that along the left-right (LR) direction, and the difference in the 2D direct distance were calculated in each slice. The mean of these C-G discrepancies was calculated for the whole prostatic urethra and for the subgroups of the superior, middle, and inferior segments in each patient (Fig. 1).

**Fig. 4:**
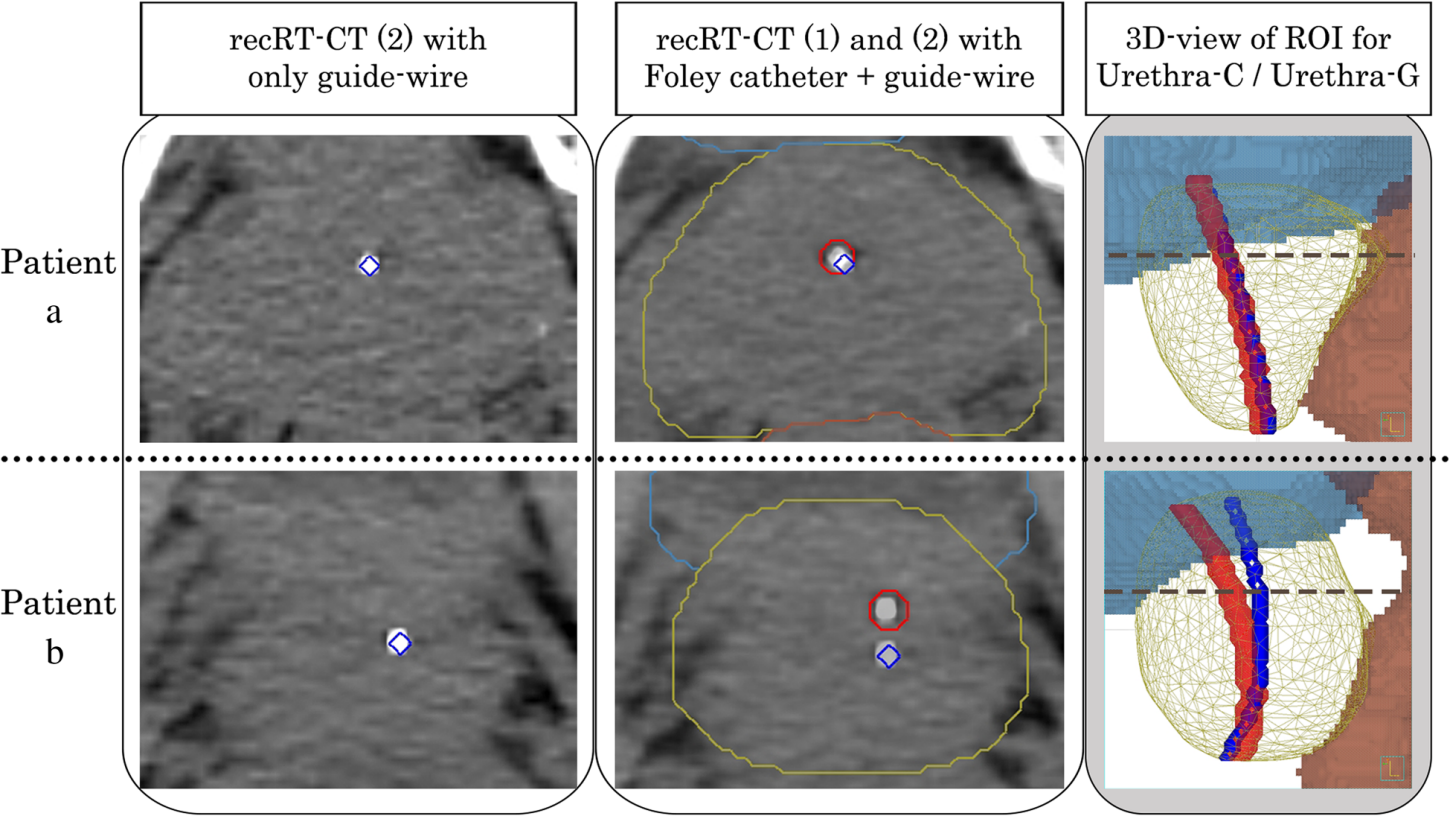
Representative patients with a small and with a large “C-G displacement”. The left image shows the recRT-CT (2) with urethra-G (blue). The middle image shows the fusion image of recRT-CT (1) and recRT-CT (2) with urethra-G (blue) and urethra-C (red). The right image shows the 3D view of urethra-G and urethra-C with the ROI for the prostate (yellow), bladder (blue), and rectum (brown). The broken line (dark blue) shows the height level of the RT-CT slices in the left and middle images. The “C-G displacement” was small throughout the whole prostatic urethra in patient **a**. On the contrary, the “C-G’ displacement” was apparent at the middle and large at the superior segment in Patient **b**

In Fig. 4, top images show a case where the “C-G discrepancy” is small, while the bottom images show a case where the “C-G discrepancy” is large.

The clinical target volume was determined based on MR images. Since the soft and thin guide-wire in the Foley catheter has little effect on the position of the catheter, the position of catheter in MR images was nearly identical to the position of the catheter in RT-CT (1). Therefore, we have not used the positional data of the catheter obtained from MR images.

### Patients and ethics

The analysis was performed retrospectively for patients who had received IMRT for localized prostate cancer without a history of prostatectomy. A total of 163 consecutively registered patients treated between December 2011 and June 2017 in one institution were eligible for inclusion in this study. The study was approved by the Institutional Review Board of Hokkaido University Hospital for Clinical Research, number 017–0475 and was also registered with UMIN (number 000036512).

### Statistics

We used Mann-Whitney’s U test to compare the distribution of the mean “C-G discrepancy” between the AP direction and LR direction in the whole urethra for all patients and each T stage (T1, T2, and T3/T4). The test was also performed at the superior, middle, and inferior segments in all patients. The variance of the mean “C-G discrepancy” was also compared by two-tailed F-test among the three segments in the AP and LR directions. The significance level was set at 0.05 in both tests.

Significant displacement, or significant “C-G discrepancy,” was defined as follows: Since the effect of the prostate motion during RT-CT (1) and RT-CT (2) had to be smaller than 1.0 mm after the rigid image registration of the three gold markers, and as the urethral catheter has a 2.0-mm outer radius, which may be assumed as < 2.0-mm inner radius, we determined that ≥3.0 mm “C-G discrepancy” should be regarded as a significant displacement.

The incidence of significant displacements was investigated slice by slice. We set four criteria to report the incidence of significant displacements, i.e., the number of patients who displayed a significant “C-G discrepancy” in one or more RT-CT slice, ≥10%, ≥20%, or ≥ 50% of the RT-CT slices in the whole prostatic urethra and called those as “one-slice criteria,” “10% criteria,” “20% criteria,” and “50% criteria”, respectively. The same criteria were used in the analysis of the incidence of significant displacements in each segment of the prostatic urethra. We used the McNemar test with the Bonferroni correction to compare the ratio of patients who met each criterion. The significance level was set at 0.0167 by the Bonferroni method, and a significant trend where the *p*-value was between 0.0167 to 0.05 was also identified. All tests were performed with JMP Pro 13.1.0 (SAS Institute, Cary, NC, USA).

## Results

Of 163 eligible patients, eight patients were excluded for the following reasons: cancer infiltration into the urethra (*n* = 2), refusal to urethral catheter insertion (*n* = 2), insertion was attempted but the insertion of the urethral catheter failed (*n* = 2), the guide-wire was accidentally removed before recording the second CT (*n* = 1), and accidental examination without the guide-wire at the second CT (*n* = 1). The 155 remaining patients were evaluated (Table 1). Patients characteristics are shown in Table 1. A total of 5531 CT slices for the 155 patients were evaluated.

**Table 1:** Characteristics of the 155 patients in this study

Number of patients / RT-CT slices		Patients / RT-CT slices	155 / 5531
Age (years old)		Median (range)	71 (53–86)
T stage (%)		1	97 (62.6)
		2	39 (25.2)
		3	18 (11.6)
		4	1 (0.6)
Gleason Score (%)		6	23 (14.8)
		7	74 (47.7)
		8	27 (17.4)
		9	30 (19.4)
		10	1 (0.6)
Number of RT-CT slices per patient (slices)		Median (range)	36 (25–54)
Prostate volume (cm3)		Median (range)	50.0 (21.4–126.7)
Length of the urethra contoured as the organ at risk	Whole urethra (mm)	Median (range)	45.5 (31.9–71.5)
	Superior segment (mm)	Median (range)	15.2 (10.1–28.0)
	Middle segment (mm)	Median (range)	15.1 (10.2–22.8)
	Inferior segment (mm)	Median (range)	15.2 (10.4–26.7)

**Fig. 5:**
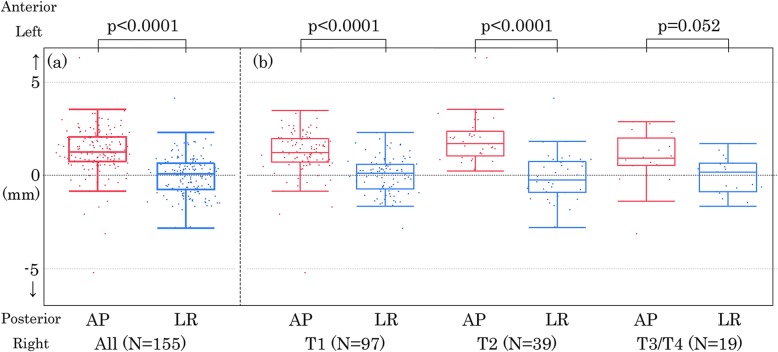
Two-dimensional scatter plots and box plots of the mean urethral displacement of the whole urethra. (**a**) All patients. (**b**) Patients with T1, T2, and T3/4 diseases, respectively. The left image (red) shows the displacement along the anteroposterior (AP) direction and the right image (blue) shows that along the left-right direction (LR). On the box plots, the center line in the box indicates the median, and the upper and lower lines of the box indicate the third and first quartiles, respectively. The maximum and minimum of the whisker indicate the “third quartile + 1.5×IQR” and the “first quartile + 1.5×IQR.” *IQR, interquartile range.* The distribution of the mean C-G discrepancy between the anteroposterior (AP) direction and left-right (LR) direction were compared by Mann-Whitney’s U test for all patients (**a**) and each T stage (**b**). A *p*-value less than 0.05 was considered statistically significant

The median (range) of the mean “C-G discrepancy” of the whole prostatic urethra was + 1.3 mm (+ 6.3 mm to − 5.2 mm) when we assumed that the anterior direction is the positive direction and the posterior is the negative direction (Table 2). Figure 5(a) shows the distribution of the mean “C-G discrepancy” in the whole urethra along the AP direction and along the LR direction. There was a statistically significant difference in the distribution of the mean “C-G discrepancy” between the two orthogonal directions (*p* < 0.0001). This suggests that the urethral catheter was displaced anteriorly more often than posteriorly, whereas the incidence of lateral displacement was similar in the left and right directions. Figure 5(b) shows that the difference in the distribution of the mean “C-G discrepancy” between the two orthogonal directions was significant among T1 (*p* < 0.0001), T2 (*p* < 0.0001) and marginally significant among T3/T4 diseases (*p* = 0.052).

**Table 2:** The median and the range of the mean C-G discrepancy

Prostate Urethra			Whole	Superior segment	Middle segment	Inferior segment
Number of patients			155	155	155	155
Anterior –Posterior direction	Median (mm)		1.3	1.4	1.4	1.2
	Range (mm)	Anterior (+)	6.3	8.3	6.4	4.9
		Posterior (−)	−5.2	−6.9	−5.2	−3.4
Lateral direction	Median (mm)		0.1	−0.2	0.1	0.1
	Range (mm)	Left (+)	4.1	7.2	3.5	3.6
		Right (−)	−2.8	−6.9	−3.1	−2.9
Direct distance	Median (mm)		2.0	2.0	1.7	1.8
	Min (mm)		0.3	0.2	0.4	0.3
	Max (mm)		7.6	11.0	7.2	5.1

The C-G discrepancy is the difference between the center of the ROI of the urethral catheter (C) and that of the guide-wire (G) on each RT-CT slice. The coordinate of the urethral catheter minus that of the guide-wire is shown as the difference

**Fig. 6:**
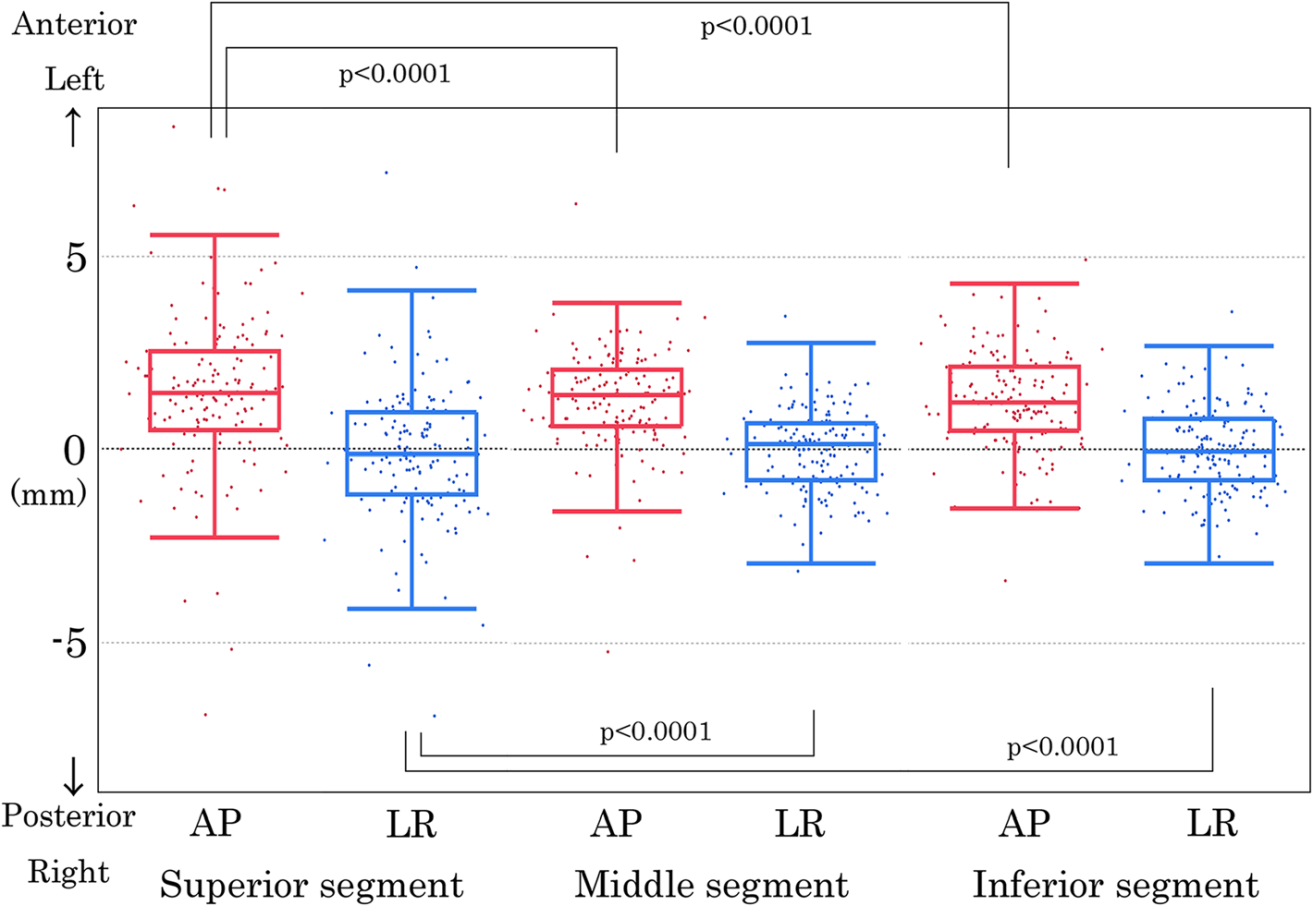
Two-dimensional scatter plots and box plots of the mean urethral displacement in each segment. The 2D scatter plots and box plots are displayed for each segment: superior (left images), middle (middle images), and inferior (right images) segments. The AP images (red) show the displacement along the AP direction and LR images (blue) show that along the LR direction. On the box plots, the center line in the box indicates the median, and the upper and lower lines of the box indicate the third and first quartiles, respectively. The maximum and minimum of the whisker indicate the “third quartile + 1.5×IQR” and “first quartile + 1.5×IQR.” *IQR, interquartile range.* The variance of the mean C-G discrepancy between the superior segment and the middle or inferior segment in the anteroposterior and left-right directions were compared by two-tailed F-test. A *p*-value less than 0.05 was considered statistically significant. AP, anteroposterior; LR, left-right

The median and range of the mean “C-G discrepancy” was compared among the superior, middle, and inferior segments (Table 2). Statistically significant differences in the distribution of the mean “C-G discrepancy” were found between the two orthogonal directions at the superior, middle, and inferior segments, respectively (*p* < 0.0001) (Fig. 6). The variance of the mean “C-G discrepancy” was significantly larger at the superior segment than at the middle (*p* < 0.0001) and inferior (*p* < 0.0001) segments both along the AP direction and along the LR direction.

Table 3 shows the incidence of the significant displacements. The number of patients who met the “one-slice criteria” was 61 (39.4%) toward the anterior direction and 84 (54.2%) in the 2D direct distance. The incidence of significant displacements toward the posterior direction where patients met the “one-slice criteria” was 5 (3.2%). The number of patients who met the “10% criteria,” “20% criteria,” and “50% criteria” toward the anterior direction was 51 (32.9%), 36 (23.2%), and 9 (5.8%), respectively. The incidences toward the left and right was less common than the anterior direction but more common than the posterior direction. The incidence of significant displacements as the 2D direct distance showed similar trends as those toward the anterior direction.

**Table 3:** The number and the proportion of patients who met the criteria

Criteria	Direction	Number of patients at risk	Whole RT-CT slices	RT-CT slices at superior segment	RT-CT slices at middle segment	RT-CT slices at inferior segment
			36 (25–54) slices	12 (8–18) slices	13 (9–18) slices	12 (8–18) slices
1 or more RT-CT slices	Anterior (+)	155	61 (39.4%)	41 (26.5%)	19 (12.3%)	24 (15.5%)
	Posterior (−)	155	5 (3.2%)	4 (2.6%)	4 (2.6%)	2 (1.3%)
	Left (+)	155	15 (10.3%)	12 (7.7%)	1 (0.6%)	3 (1.9%)
	Right (−)	155	16 (9.7%)	15 (9.7%)	3 (1.9%)	1 (0.6%)
	2D distance	155	84 (54.2%)	65 (41.9%)	40 (25.8%)	38 (24.5%)
10% or more RT-CT slices	Anterior (+)	155	51 (32.9%)	39 (25.2%)	17 (11.0%)	22 (14.2%)
	Posterior (−)	155	4 (2.6%)	4 (2.6%)	3 (1.9%)	1 (0.6%)
	Left (+)	155	11 (7.1%)	11 (7.1%)	1 (0.6%)	3 (1.9%)
	Right (−)	155	7 (4.5%)	11 (7.1%)	2 (1.3%)	1 (0.6%)
	2D distance	155	77 (49.7%)	62 (40.0%)	37 (23.9%)	34 (21.9%)
20% or more RT-CT slices	Anterior (+)	155	36 (23.2%)	38 (24.5%)	14 (9.0%)	18 (11.6%)
	Posterior (−)	155	4 (2.6%)	4 (2.6%)	3 (1.9%)	1 (0.6%)
	Left (+)	155	7 (4.5%)	8 (5.2%)	1 (0.6%)	2 (1.3%)
	Right (−)	155	5 (3.2%)	10 (6.5%)	2 (1.3%)	1 (0.6%)
	2D distance	155	56 (36.1%)	61 (39.4%)	31 (20.0%)	29 (18.7%)
50% or more RT-CT slices	Anterior (+)	155	9 (5.8%)	27 (17.4%)	9 (5.8%)	12 (7.7%)
	Posterior (−)	155	1 (0.6%)	4 (2.6%)	1 (0.6%)	1 (0.6%)
	Left (+)	155	0 (0.0%)	4 (2.6%)	1 (0.6%)	1 (0.6%)
	Right (−)	155	1 (0.6%)	9 (5.8%)	0 (0.0%)	1 (0.6%)
	2D distance	155	20 (12.9%)	51 (32.9%)	18 (11.6%)	22 (14.2%)

The criteria were the number and the ratio of RT-CT slices in each patient which showed the calculated coordinate of the urethral catheter minus that of the guide-wire as 3.0 mm or larger in the corresponding direction

There were 41 (26.5%), 19 (12.3%), and 24 (15.5%) patients who met the “one-slice criterion” toward the anterior direction in the superior, middle, and inferior segments. The incidence of significant displacements toward the posterior direction in patients who met the “one-slice criteria” was 4 (2.6%), 4 (2.6%), and 2 (1.3%) in each segment. The largest number of patients who met the “one-slice criterion” in the 2D direct distance in the superior, middle, and inferior segments was 65 (41.9%), 40 (25.8%), and 38 (24.5%), respectively.

There were 38 (24.5%), 14 (9.0%), and 18 (11.6%) patients who met the “20% criterion” toward the anterior direction in the superior, middle, and inferior segments, respectively. There were 61(39.4%), 31(20.0%), 29(18.7%) patients who met the “20% criterion” in the 2D direct distance in each segment. There were 27 (17.4%) and 51 (32.9%) patients who met the “50% criterion” toward the anterior direction and in the 2D direct distance in the superior segment.

**Fig. 7:**
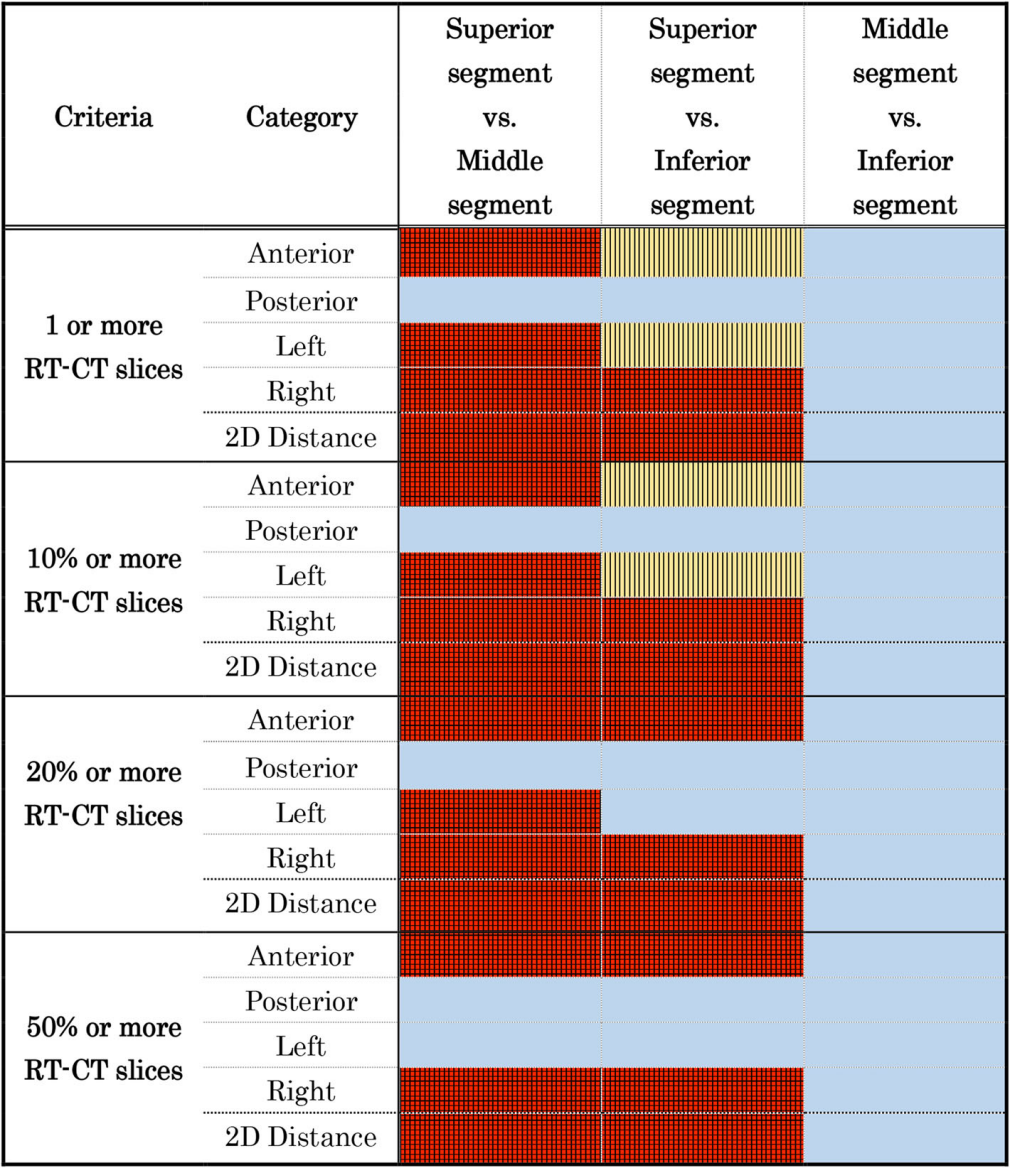
Results of the McNemar test of the number of patients who met the criteria Detail of the criteria is shown in the left column. The McNemar test was performed between each of the different segments of the prostate urethra. The significance level was set at *p* < 0.0167 (red plus lattice-patterned area), significant trends at 0.0167 ≦ *p* < 0.05 (yellow plus stripe-patterned area), and absence of significance at ≦0.05 (light blue area) by the Bonferroni method

The results of the statistical comparison using the McNemar test for the incidence of significant displacements between different segments are shown in Fig. 7. Significantly larger proportions of patients met the criterion for anterior displacement at the superior segment than at the middle segment. The anterior displacement at the superior segment was marginally or significantly more frequent than at the inferior segment. Significantly larger proportions of patients met the criterion for lateral displacement, both in the left and right directions, and at the superior segment rather than at the middle segment. The lateral displacement at the superior segment was marginally or significantly more frequent than at the inferior segment. No significant differences were shown in the comparison between the middle and inferior segments at any direction and in the direct distance. The results of the larger difference in the 2D direct distance reflect the results along the two orthogonal directions.

## Discussion

To the best of our knowledge, the present study is the first to investigate the discrepancy between the three-dimensional position of the urethra with a Foley catheter and that of the urethra with a guide-wire. The urethra with a catheter was significantly displaced from the urethra with a guide-wire in many occasions. The number of patients who experienced large anterior displacement with the catheter was significantly larger at the superior segment than at the middle or inferior segment.

This leads to the question of whether it is possible to describe the “urethra with a guide-wire” as the better “surrogate urethra” than the “urethra with a Foley catheter.” The significant C-G discrepancy suggests that the Foley catheter itself displaces the urethral position because of its rigidity. The guide-wire would not influence the actual position of the urethra at rest so much by its rigidity and thickness. However, the guide-wire may be too thin to represent the center of the urethra since it can move in the urethral lumen, which has an “inverted V” shape in MRI micturating urethrography [26]. Therefore, it is not possible to know whether the guide-wire alone is better than the Foley catheter to represent the actual position of the urethra at rest in general.

Merrick et al. used the catheter or the geometric center of the prostate to estimate the urethral position [27]. Waterman et al. used the geometric center on CT scans as a “surrogate urethra,” and Bucci et al. used the surrogate position deviated from the geometrical center on CT scans [20, 28]. Lee et al. have suggested that the Foley catheter is located anterior to the geometric center with the mean discrepancy of 12, 4, and 2.5 mm at the superior, middle, and inferior segments of the prostatic urethra, respectively, after the 125-I implantation [21]. Nilsson et al. investigated the discrepancy of the Foley catheter and the geometric center by ultrasound and found that catheter displacement was mostly toward the anterior direction [29]. These studies suggest that the “urethra with a Foley catheter” is often located anterior to the geometric center of the prostate. In addition, the results may have suggested that the Foley catheter in the urethra itself has changed the actual position of the urethra at rest. A recent study by Litzenberg et al. showed that removing the Foley catheter from the urethra caused significant prostate rotation [12].

The PRV for the prostate urethra in USRT should take into account urethra displacements estimated in the present study. While the incidence of significant displacements in the 2D direct distance at the middle and inferior segments was smaller than that in the superior segment, it was not negligible. Therefore, we are now considering that only when the C-G discrepancy is < 2.0 mm that the positions of the urethra-G and urethra-C are sufficiently reliable to determine the PRV of the urethra. The PRV margin for urethra-G is set at 2.0 mm considering a possible displacement of the guide-wire in the urethral lumen. If the C-G discrepancy is > 2.0 mm, we cannot recommend using urethra-G or urethra-C to make the PRV. Although this is not a perfect solution, this study showed that it is preferable to use both RT-CT (1) and RT-CT (2) to determine the PRV for the urethra at rest, and this would provide more confidence in the treatment planning for USRT. As a result, many patients are not good candidates for USRT because we cannot make a reliable PRV at the superior segment.

Although the “urethra with a Foley catheter” is referred to be used as the ground truth in the GEC/ESTRO-EAU recommendations [22], the present study suggested that the “urethra with a Foley catheter” is not a suitable “surrogate urethra” in general. Recently, population-based prediction methods of the prostate urethral position are published using CT image set or ultrasound image set [30, 31]. Acosta et al. proposed a sophisticated method but used the urethra with a Foley catheter as the ground truth in their analysis [30]. Halperin et al. suggested the risk of dislocation of the urethra with the catheter in brachytherapy using ultrasound images, but it is difficult to use ultrasound image for external beam USRT [31].

In the future, with the use of advanced MRI in the treatment planning or MRI-guided treatment, the urethra may be visualized well [32]. Rai et al. have recently shown that dynamic MRI during micturition can visualize the actual position of the “urethra without a Foley catheter” in a patient [26]. One may expect that MRI micturating urethrography is suitable to find the actual position of the urethra at rest, but the method requires more definitive attention to anatomical differences in the positions of the bladder and prostate during micturition when compared with the position at rest.

This study has several shortcomings. First, we have not taken MRI in the identification of the urethra without Foley catheter to avoid prolonging the examination time for each patient. Second, the C-G discrepancy can change daily even in the same patient. The daily variation of the C-G discrepancy in the same patient should be investigated in more detail in future study. The 2-min difference between RT-CT with and without the Foley catheter has the potential to change the location of the prostate and urethra. The urethra may not return to the actual position without the catheter from its position with the catheter within the 2-min interval. The filling of the bladder by urine or movement of gas in the rectum may also change the position of the prostate within the 2-min interval. However, since the position of the prostate was recorded three-dimensionally using three fiducial markers between the initial CT and the second CT, the influence of the motion of the prostate gland would be minimal.

## Conclusions

Radiation oncologists and medical physicists have to be aware that the urethral position with the Foley catheter is different from the urethral position with thin and soft guide-wire at ≥3.0 mm in a proportion of patients with prostate cancer. This should be taken into account for the PRV of the urethra to ensure precise radiotherapy such as in urethra-sparing radiotherapy. When individualized precise treatment planning is required, such as in USRT, it is advantageous to use both the Foley catheter and the guide-wire in the same patient in the treatment planning. Only when the C-G discrepancy is < 2.0 mm in each of the RT-CT slices that the use of either of urethra-G or urethra-C is acceptable with some confidence when determining the PRV.

## Data Availability

The datasets analyzed during the current study are available from the corresponding author on reasonable request.

## Abbreviations

2D: Two-dimensional
3D: Three-dimensional
AP: Antero-posterior
C: Urethral catheter
CT: Computed tomography
F: French
G: Guide-wire
GI: Gastro-intestinal
GU: Genito-urinary
IG-IMRT: Image-guided IMRT
IMRT: Intensity modulated radiation therapy
LR: Left-right
MRI: Magnetic resonance imaging
PRV: Planning organ at risk volume
recRT-CT: reconstructed RT-CT
ROI: Region of interest
RT-CT: CT for radiation treatment planning
RTRT: Real-time tumor-tracking radiotherapy
SD: Standard deviation
USRT: Urethra-sparing radiotherapy
